# Enhanced expression of epithelial sodium channels causes salt-induced hypertension in mice through inhibition of the α_2_-isoform of Na^+^, K^+^-ATPase

**DOI:** 10.14814/phy2.12383

**Published:** 2015-05-19

**Authors:** Frans H H Leenen, Xiaohong Hou, Hong-Wei Wang, Monir Ahmad

**Affiliations:** University of Ottawa Heart InstituteOttawa, Ontario, Canada

**Keywords:** Angiotensin II, brain, endogenous ouabain, Liddle syndrome

## Abstract

Knockout of the Nedd4-2 gene in mice results in overexpression of epithelial sodium channels (ENaC) on the plasma membrane in the kidney, choroid plexus and brain nuclei. These mice exhibit enhanced pressor responses to CSF [Na^+^] as well as dietary salt-induced hypertension which both can be blocked by central infusion of the ENaC blocker benzamil. Functional studies suggest that ENaC activation in the CNS results in release of endogenous ouabain (EO) and inhibition of the *α*_2_-isoform of Na^+^, K^+^-ATPase. To test this concept more specifically, we studied Nedd4-2^−/−^ mice expressing the ouabain-resistant 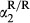-isoform of Na^+^, K^+^-ATPase. Intracerebroventricular (icv) infusion of Na^+^-rich aCSF (225 mmol/L Na^+^ at 0.4 *μ*L/min) increased MAP by 10–15 mmHg in wild-type mice and by 25–30 mmHg in Nedd4-2^−/−^ mice, but by only ~5 mmHg in 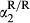 and in 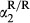/Nedd4-2^−/−^ mice. Icv infusion of EO-binding Fab fragments also blocked the BP response in Nedd4-2^−/−^ mice. In Nedd4-2^−/−^ mice, 8% high-salt diet increased MAP by 25–30 mmHg, but in 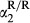/Nedd4-2^−/−^ mice, it increased by only 5–10 mmHg. In contrast, Nedd4-2^−/−^ or 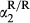 did not affect the hypertension caused by sc infusion of Ang II. These findings substantiate the concept that enhanced ENaC activity causes salt-induced pressor responses mainly through EO inhibiting the *α*_2_-isoform of Na^+^, K^+^-ATPase in the brain.

## Introduction

Functional studies have shown that a chronic increase in cerebrospinal fluid (CSF) [Na^+^] by either intracerebroventricular (icv) infusion of Na^+^-rich artificial CSF (aCSF) or high-salt diet in genetic models of salt-sensitive hypertension activates in the brain a mineralocorticoid receptor (MR) – epithelial sodium channel (ENaC) – endogenous ouabain (EO) pathway which plays an important role in the CSF [Na^+^]↑ or high-salt diet-induced sympathetic hyperactivity and hypertension (for review Blaustein et al. 2012; Gabor and Leenen 2012). This concept relies to a large extent on blockade studies using central infusions of pharmacological agents such as benzamil to block ENaC or antibody Fab fragments to bind EO, none of which – particularly benzamil (Kleyman and Cragoe 1988; Drummond 2009) – can be considered truly specific for their target. In contrast, genetically engineered mice provide a very specific approach. Lingrel et al. developed a mouse strain (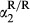) expressing a cardiac glycoside-insensitive *α*_2_-isoform of Na^+^, K^+^-ATPase which does not respond to ouabain (Dostanic et al. 2003, 2005; Van Huysse et al. 2011). These mice show no increase in blood pressure (BP) in response to central infusion of Na^+^-rich aCSF or ouabain (Van Huysse et al. 2011), indicating that inhibition of the *α*_2_-isoform in the brain mediates the sympatho-excitatory and pressor responses to EO or ouabain infused into the brain.

Ubiquitination of ENaC by Neural precursor cell expressed and developmentally downregulated 4-2 protein (Nedd4-2) facilitates the endocytosis of ENaC from the plasma membrane (Raikwar and Thomas 2008). Knockout of the Nedd4-2 gene in mice results in overexpression of ENaC not only in the kidney (Shi et al. 2008) but also in the choroid plexus and brain nuclei such as the SON and PVN (Van Huysse et al. 2012). The Nedd4-2^−/−^ mouse serves as a model of Liddle syndrome (Hansson et al. 1995). These mice exhibit enhanced pressor responses to icv infusion of Na^+^-rich aCSF (Van Huysse et al. 2012), an increase in CSF[Na^+^] on 8% high-salt diet (Van Huysse et al. 2012) and salt-dependent hypertension (Shi et al. 2008; Van Huysse et al. 2012). Central infusion of benzamil markedly inhibits both the enhanced pressor response to Na^+^-rich aCSF and the high-salt diet-induced hypertension (Van Huysse et al. 2012), consistent with an important functional role of ENaC overexpression in the brain. We hypothesized that these BP responses to enhanced ENaC activity involve release of EO and resulting inhibition of the high-affinity *α*_2_-isoform. If so, these pressor responses should be absent in Nedd4-2^−/−^ mice expressing the 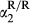-isoform. We, therefore, by cross-breeding, generated an 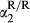/Nedd4-2^−/−^ mouse-line and in these animals assessed by telemetry BP and HR responses to icv infusion of Na^+^-rich aCSF as well as BP and HR responses to 8% high-salt diet. To assess whether the observed pattern of response is specific for sodium, we also evaluated responses to subcutaneous (sc) infusion of Angiotensin II (Ang II).

## Methods

### Ethical approval

All studies were approved by the University of Ottawa Animal Care Committee and conform to the Guide for the Care and use of Laboratory Animals published by the National Institutes of Health (8th Edition, 2011).

### Animals

**Figure 1:**
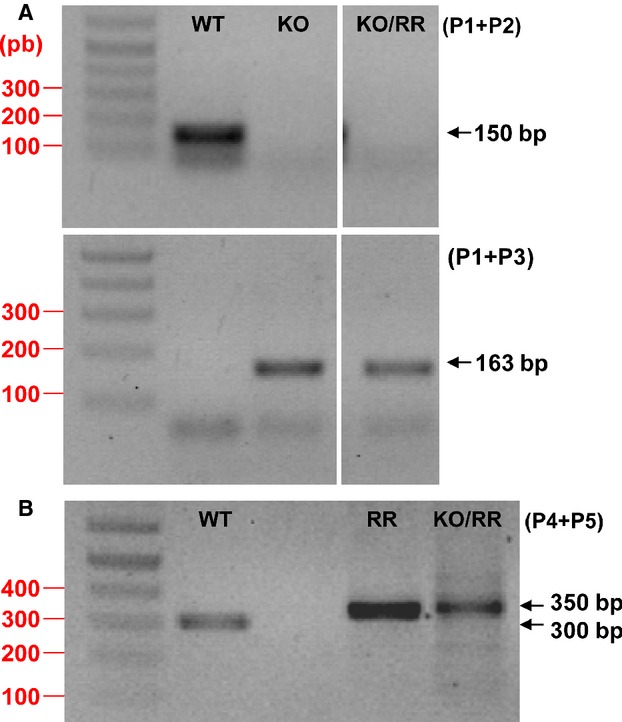
Genotyping analysis. (A) Primer 1 and 2 amplification from genomic DNA generated the wild-type 150 bp fragment. P1 and 3 resulted in 163 bp fragment for the Nedd4-2 knockout allele. (B) Primer 4 and 5 produced ~300 bp fragment for wild type and ~350 bp for 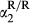. WT: wild type. KO: Nedd4-2 knockout. RR: 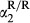. KO/RR: 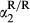/Nedd4-2^−/−^.

The 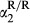 mouse-line was obtained from Dr. Jerry Lingrel at the University of Cincinnati, and the Nedd4-2^−/−^ mouse-line from Dr. Baoli Yang at the University of Iowa. Breeding colonies for both mouse-lines and their respective wild-type (WT) controls have been established at the University of Ottawa Heart Institute. The 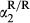/Nedd4-2^−/−^ mouse was obtained by cross-breeding until mice homozygous for both were generated (Fig.1). Male mice, 9–12 weeks of age, were used because of their larger size. Mice were housed in group cages before surgery, then individually postoperatively, in a temperature controlled environment with a 12:12 h light-dark cycle. Water and standard mouse chow (0.3% NaCl) were provided ad libitum, except when a high-salt diet (8% NaCl, TD.92012 from Harlan Laboratories, Madison, WI) was substituted for the standard chow. For all surgeries, mice were anesthetized with 2% isoflurane in oxygen. For pain relief, slow-release buprenorphine (0.2 mg/kg) was injected subcutaneously 1/2 h before surgery, which provides adequate analgesia for 3 days.

For genotyping, genomic DNA was extracted from tail samples obtained at 3 weeks of age. To distinguish the knockout versus WT alleles of the Nedd4-2 gene, dual PCR reactions were performed in parallel for each sample, each using a separate set of primers in which the reverse primer was specific for the WT or −/− allele (Van Huysse et al. 2012). A forward primer (P1, 5′-TGAGCTCATTGCTTCACTTCC-3′) and the reverse primer (P2, 5′-TTCATGCTCGAAGCCTTAGC-3′) resulted in a 150-bp amplicon to identify the WT allele. P1 and the reverse primer P3 (5′-TTTGTGAGGACAGCCTCTAGC-3′) only produced a product of amplifiable size (163 bp) for the knockout allele. To identify “R” and WT alleles at the *α*_2_ locus, a single allele-specific PCR reaction was performed using forward primer P4 (5′- TCAGCTGTGGCTCCACGTGGG-3′) and reverse primer P5 (5′- GCATGGGGGATTGGGGGATTA-3′) (Van Huysse et al. 2011). The PCR reaction generated ~300 and 350 bp products for the wild type and 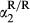 genotypes, respectively (Fig.1).

### Surgeries

Seven days before icv infusions, a 23G guide cannula was implanted into a lateral brain ventricle with stereotaxic coordinates: 0.1 mm anterior; 1.0 mm lateral and 1.5 mm ventral to lambda. The cannula was secured with dental acrylic cement and the skin was closed. In the same surgical session, the catheter tip of a TAIIPA-C10 transmitter (Data Sciences International, St. Paul, MN) was inserted into the left carotid artery and secured with double ligatures. The transmitter itself was implanted subcutaneously. The signal levels were then tested and the animals placed in recovery chambers. For high-salt diet experiments, the transmitter was implanted 10–12 days before the start of diet.

### Experimental protocols

#### Icv infusion of Na^+^-rich aCSF

All studies were performed in a quiet room in the morning. The transmitters were turned on, and after a rest period of 30 min, baseline MAP and HR were recorded for 30 min. Icv infusion of Na^+^-rich aCSF (225 mmol/L Na^+^) was then started at 0.4 *μ*L/min and continued for 60 min. This rate of infusion of sodium causes modest increases in BP and HR in WT mice (Hou et al. 2009), whereas infusion of regular aCSF at the same rate does not increase BP or HR (Hou et al. 2009; Van Huysse et al. 2012). The average BP and HR for each 30 sec interval were calculated. In one additional group of Nedd4-2^−/−^ mice, 100 *μ*g DigiFab (EO-binding antibody Fab fragments; Paladin Labs, Montreal, Quebec, Canada) was infused over 30 min, and then 100 *μ*g DigiFab combined with 225 mmol/L of Na^+^-rich aCSF were infused for 60 min.

#### High-salt diet

In mice on regular-salt diet, the transmitters were turned on, and baseline BP and HR recorded for 3 days. Mice were then placed on 8% high-salt diet for 12–14 days. BP and HR were recorded at the beginning of every hour for 2 min and averages used for statistical analysis.

#### Sc infusion of Ang II

7–10 days after telemetry transmitter implantation baseline BP and HR were recorded for 2–3 days followed by sc implantation of osmotic minipumps (model 1002; Alzet, Palo Alta, CA) prefilled with Ang II diluted in sterile saline. To assess for dose-dependent interactions, Ang II was infused at the low dose of 200 ng/kg/min as well as the high dose of 1000 ng/kg/min (Zimmerman et al. 2004). Ang II concentrations of 1.2 and 6 mg/mL in the pump were calculated based on the average weight of the mice (25 g), minipump flow rate of 0.25 *μ*L/h and infusion duration of 14 days. Prefilled pumps were stored in sterile 0.9% saline at room temperature to ensure a constant pumping rate at the time of implantation. Control mice underwent sham surgery.

### Statistical analysis

All values are expressed as mean ± SE. Between-group changes in blood pressure (Δ BP) and heart rate (Δ HR) from baseline (by high-salt diet or Na^+^-rich aCSF) were compared for areas under the curve by one-way ANOVA. Areas under the curve were calculated with Sigma Plot. When ANOVA detected significant differences between groups, post hoc comparisons were made by Student–Newman–Keuls test. For BP and HR changes from baseline, one-way ANOVA with repeated measures was performed. Statistical significance was defined as *P* < 0.05.

## Results

**Figure 2:**
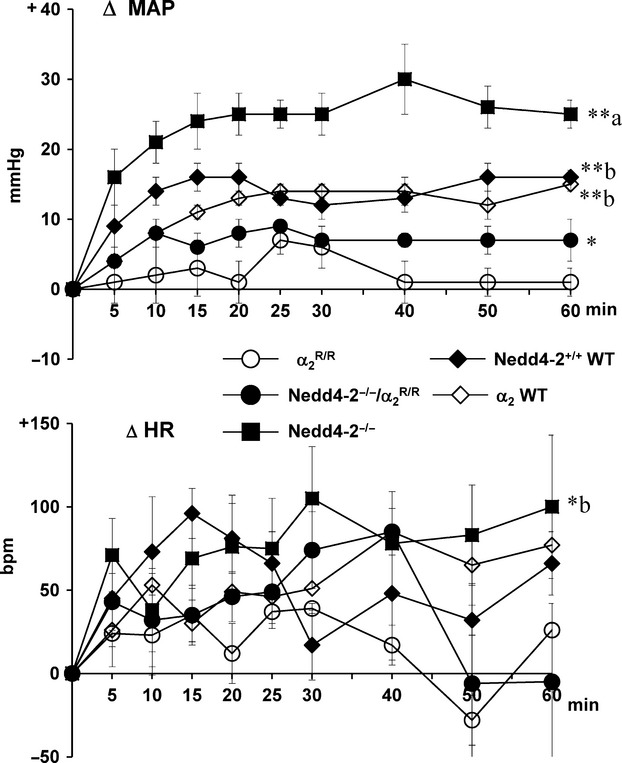
Enhanced BP and HR responses to icv infusion of Na^+^-rich aCSF (225 mM Na^+^) at 0.4 *μ*L/min in Nedd4-2^−/−^ mice versus wild-type (WT) controls but not in 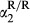/Nedd4-2^−/−^ mice. Blood pressures and heart rates were averaged over 5–15 min intervals. Values represent the mean ± SEM of the changes from baseline. **P < 0.001; *P < 0.05 versus baseline. Between-group changes were compared for areas under the curve:

In the WT groups, the 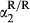, Nedd4-2^−/−^ and the 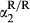/Nedd4-2^−/−^ mice, baseline MAPs were similar at around 110–120 mmHg and baseline HR in the 500–600 bpm range (Table1). Infusion of Na^+^-rich aCSF increased MAP by 10–15 mmHg (*P* < 0.0001) in WT mice (Fig.2). In contrast, MAP did not increase significantly in the 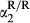 group and increased significantly more (*P* < 0.0001 vs. other groups) up to 25–30 mmHg in the Nedd4-2^−/−^ group. In the 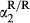/Nedd4-2^−/−^ group, Na^+^-rich aCSF increased MAP by only 5–10 mmHg (*P* < 0.05 vs. baseline), which was not significantly different from the change in the 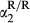 group (Fig.2).

**Table 1:** Baseline MAP and HR in the different groups of mice used for icv infusion of Na^+^-rich aCSF (data in Fig.2), or for sc infusion of Ang II at 200 or 1000 ng/kg/min (data shown in Fig.5).

Groups	MAP (mmHg)	HR (bpm)
ICV infusion studies		
WT	121 ± 8	511 ± 39
WT	119 ± 5	568 ± 25
Nedd4-2^−/−^	125 ± 8	559 ± 43
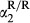	128 ± 3	556 ± 19
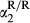 /Nedd4-2^−/−^	124 ± 7	533 ± 34

Groups	MAP (mmHg)		HR (bpm)	
	Low Dose	High Dose	Low Dose	High Dose
Sc infusion of Ang II studies (daytime values)				
WT	107 ± 2	115 ± 6	525 ± 15	525 ± 15
Nedd4-2^−/−^	107 ± 6	112 ± 4	508 ± 36	545 ± 10
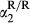	–	125 ± 4	–	557 ± 7
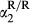 /Nedd4-2^−/−^	107 ± 6	113 ± 5	464 ± 22	481 ± 18

Values represent the mean ± SEM. For n/group see legends to Figs.2 and 5.

BP: *α*_2_WT: 823 ± 54 (*n* = 4); Nedd4-2^+/+^WT: 634 ± 67 (*n* = 4); Nedd4-2^−/−^: 1401 ± 172 (*n* = 5); 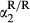: 140 ± 72 (*n* = 5), and Nedd4-2^−/−^/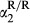: 410 ± 64 (*n* = 5). 

*F*-value 21.8, *P* < 0.0001.

HR: WT: 3314 ± 459 (*n* = 8); Nedd4-2^−/−^: 4784 ± 1342 (*n* = 5); 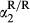: 987 ± 533 (*n* = 5), and Nedd4-2^−/−^/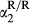: 2468 ± 585 (*n* = 5).

*F*-value 3.5, *P* = 0.04.

^a^*P* < 0.0001 versus others; ^b^*P* < 0.05 versus 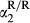.

Na^+^-rich aCSF significantly (*P* < 0.05 vs. baseline) increased HR in the Nedd4-2^−/−^group by 90–110 bpm and in the other groups by only 20–50 bpm (*P* = 0.07–0.1). No significant (*P* = 0.15) between-group responses in HR were found (Fig.2).

**Figure 3:**
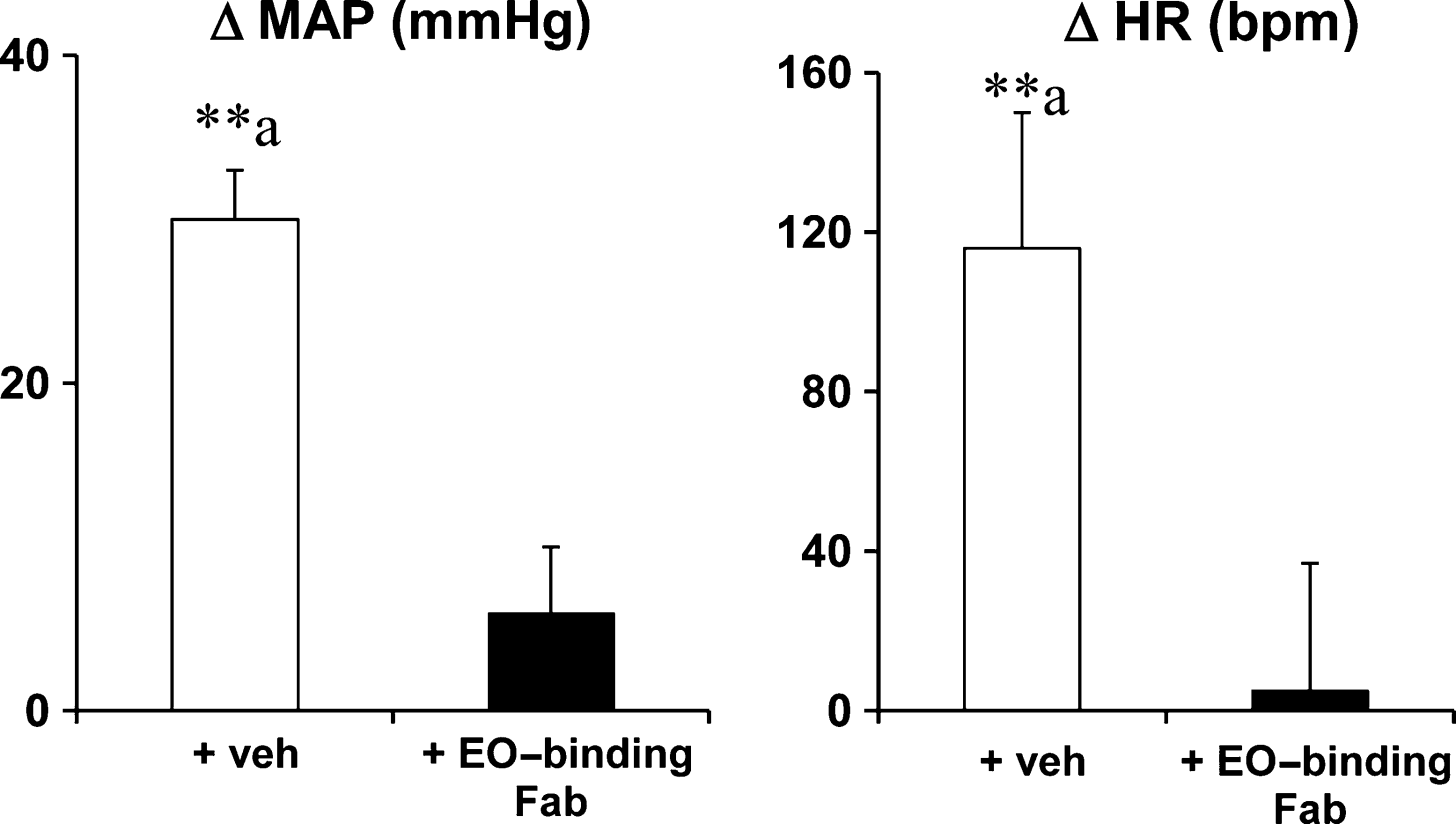
MAP and HR responses to icv infusion of Na^+^-rich aCSF (225 mM Na^+^) at 0.4 *μ*L/min in Nedd4-2^−/−^ mice are blocked by icv infusion of EO-binding antibody Fab fragments. Values represent the peak changes (mean ± SEM) from baseline (*n* = 4 and 5/group). **P < 0.001 versus baseline; ^a^*P* < 0.001 versus group with Fab fragments.

In Nedd4-2^−/−^ mice on regular-salt diet, icv infusion of EO-binding antibody Fab fragments did not change resting MAP (121 ± 6 vs. 122 ± 5 mmHg) or HR (612 ± 47 vs. 607 ± 44 bpm). When combined with Fab fragments, icv infusion of Na^+^-rich aCSF in these mice caused only a minor (NS) increase in MAP by 3–5 mmHg, and no change in HR (−10 to +30 bpm). See Fig.3 for peak changes.

**Figure 4:**
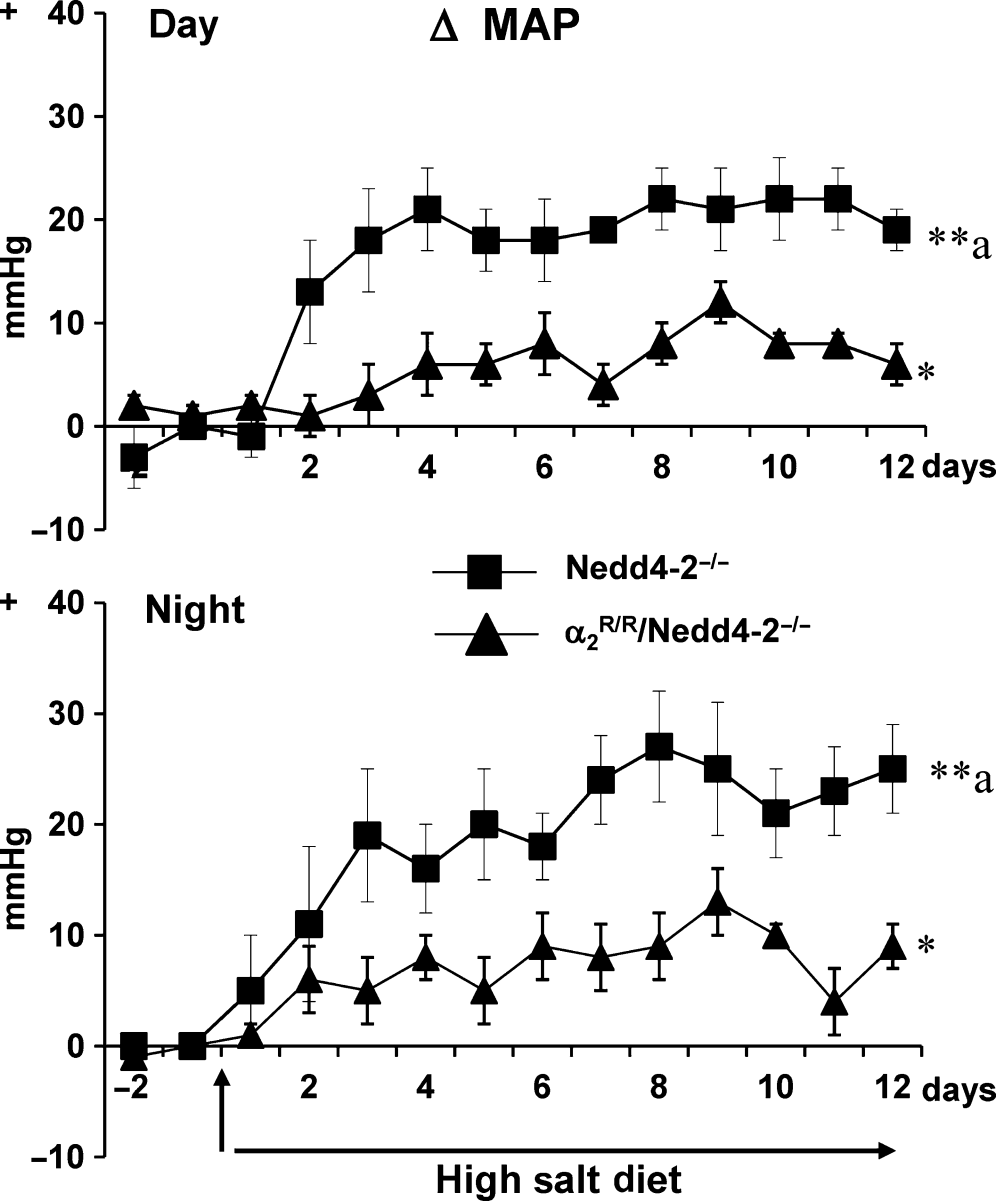
Effects of high-salt diet (8% NaCl) on mean arterial pressure (MAP) during the day (7:00 AM to 6:00 PM) and night (7:00 PM to 6:00 AM) in Nedd4-2^−/−^ (*n* = 4) and in 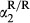/Nedd4-2^−/−^ mice (*n* = 6). The baseline average MAP was 106 ± 1 and 116 ± 1 mmHg for the day, and 116 ± 4 and 126 ± 2 mmHg for the night in the Nedd4-2^−/−^ and 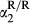/Nedd4-2^−/−^ mice, respectively. Values represent the mean ± SEM of the changes from baseline (day −1 to 0). **P < 0.001; *P < 0.05 versus baseline. Between-group changes were compared for areas under the curve:

In Nedd4-2^−/−^ mice, 8% high-salt diet increased day and night time MAP by 25–30 mmHg and HR by 30–40 bpm after 6–8 days (Fig.4). In contrast in the 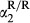/Nedd4-2^−/−^ mice, high-salt diet increased day and night time MAP (Fig.4) by only 5–10 mmHg (*P* < 0.05 vs. baseline) and did not affect HR (data not shown).

BP Day: Nedd4-2^−/−^: 223 ± 33 and Nedd4-2^−/−^/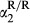: 74 ± 11.

*F*-value 7.2, *P* = 0.03.

BP Night: Nedd4-2^−/−^: 238 ± 52 and Nedd4-2^−/−^/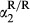: 84 ± 18.

 *F*-value 9.0, *P* = 0.02.

^a^p<0.05 versus 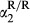/Nedd4-2^−/−^.

**Figure 5:**
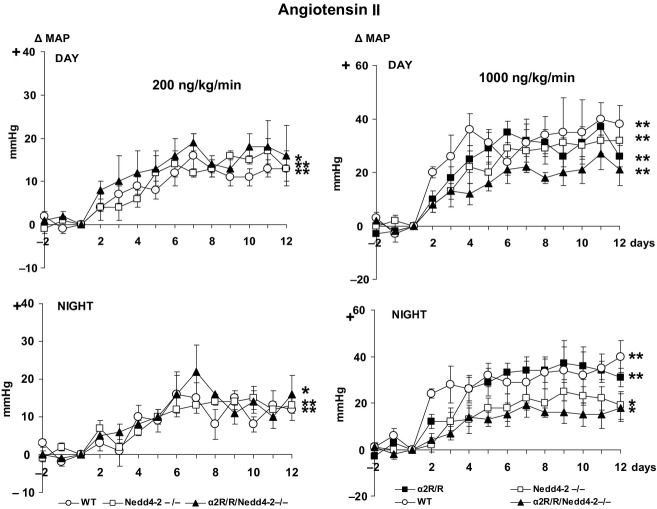
Effects of sc infusion of Ang II at 200 ng/kg/min (left panels) and at 1000 ng/kg/min (right panels) on mean arterial pressure (MAP) during the day and night in wild-type (WT) controls (*n* = 6 and 5), Nedd4-2^−/−^ (*n* = 4 and 6), 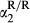 (*n* = 7 for high dose only), and 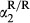/Nedd4-2^−/−^ (*n* = 4 and 4). Values represent the mean ± SEM of the changes from baseline (day −1 to 0). **P < 0.001; *P < 0.05 versus baseline. Between-group changes were compared for areas under the curve:

Sc infusion of Ang II at the rate of 200 ng/kg/min caused gradual modest increases in day and night time MAP by 10–15 mmHg, which was similar in WT, Nedd4-2^−/−^ and 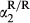/Nedd4-2^−/−^ mice (Fig.5). Ang II at the rate of 1000 ng/kg/min increased MAP in WT mice by 30–40 mmHg. The increases were similar in the 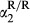 mice, but tended (*P* = 0.10) to be less in the Nedd4-2^−/−^ and 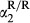/Nedd4-2^−/−^ mice at night (Fig.5). Heart rate showed only minor, nonsignificant changes with either dose of Ang II (data not shown).

BP Day: *low dose* – WT:122 ± 14, Nedd4-2^−/−^: 109 ± 11 and Nedd4-2^−/−^/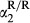: 172 ± 44.

*F*-value 1.4, *P* = 0.29.

*High dose* – WT:385 ± 71, Nedd4-2^−/−^: 316 ± 53, 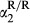: 266 ± 58 and 

Nedd4-2^−/−^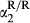: 189 ± 23.

 *F*-value 1.4, *P* = 0.20.

BP Night: *low dose* – WT:128 ± 18, Nedd4-2^−/−^: 121 ± 19 and Nedd4-2^−/−^/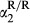: 147 ± 15.

 *F*-value 0.5, *P* = 0.63.

*High dose* – WT:407 ± 92, Nedd4-2^−/−^: 227 ± 45, 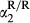: 277 ± 57 and 

Nedd4-2^−/−^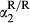: 161 ± 36.

*F*-value 2.4, *P* = 0.10.

## Discussion

This study demonstrates that the enhanced pressor responses to increased CSF[Na^+^] and high-salt diet-induced hypertension in Nedd4-2^−/−^ mice are largely absent in the presence of the ouabain-resistant *α*_2_-isoform of Na^+^, K^+^-ATPase. These findings support the concept that enhanced ENaC activity causes salt-induced pressor responses through EO inhibiting the *α*_2_-isoform in the brain.

Sodium transport proteins such as ENaC and Na^+^, K^+^-ATPase are co-expressed in the kidneys where they play a critical role in regulation of sodium balance by regulating sodium re-absorption across the nephron. Enhanced renal ENaC activity increases renal sodium and water re-absorption, and it is commonly assumed that this is the primary mechanism leading to salt-induced hypertension by high-ENaC activity (Ronzaud and Staub 2014). However, we recently demonstrated that the hypertension caused by high-salt diet in mice with enhanced ENaC activity may actually be due to increased sodium levels in the CSF and enhanced pressor responses to CSF sodium (Van Huysse et al. 2012). Although in the kidney and choroid plexus ENaC and Na^+^, K^+^-ATPase mediate Na^+^-transport across the cell membrane (Amin et al. 2005, 2009; Gonzalez-Vicente and Garvin 2013), neuronal ENaC appears to be part of a functional neuromodulatory pathway involving aldosterone -MR -ENaC –EO (Gabor and Leenen 2012). Both MR and ENaC exhibit high expression in magnocellular neurons of the PVN and SON (Amin et al. 2005; Ito et al. 2010; Wang et al. 2010; Teruyama et al. 2012), and in these neurons ENaC currents contribute to the resting membrane potential and modulate neuronal activity (Teruyama et al. 2012). As reviewed recently (Gabor and Leenen 2012), aldosterone may via MR and ENaC increase EO release from magnocellular neurons (Yoshika et al. 2011). From a functional perspective, the resulting sympatho-excitatory and pressor responses to central sodium or aldosterone can be prevented by antibody Fab fragments binding EO, indicating that brain EO mediates these responses. The present studies show that these Fab fragments also block the enhanced pressor responses to CSF [Na^+^] in Nedd4-2^−/−^ mice further supporting the concept that ENaC activation in the CNS leads to local EO release. The downstream response to EO depends on inhibition of Na^+,^ K^+^-ATPase (Blaustein et al. 2012). In the brain, the *α*_3_-isoform is specific for neurons and has a greater affinity for ouabain than the *α*_2_-isoform which is mainly present in glia. In 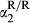 mice, specific [^3^H] ouabain binding in the brain is reduced by ~30%, and the remaining ouabain-binding reflects persistent binding to the *α*_3_-subunit (Dostanic et al. 2003). Surprisingly, the *α*_2_ rather than the *α*_3_-isoform mediates the pressor responses to icv infusions of Na^+^-rich aCSF or of ouabain (Van Huysse et al. 2011). The present study shows that EO and the ouabain-sensitive *α*_2_-isoform are also essential for the enhanced pressor responses to central sodium in Nedd4-2^−/−^ mice, since the icv infusion of EO-binding antibody Fab fragments prevents most of the pressor responses, and the BP responses are markedly less in the 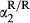/Nedd4-2^−/−^ mice. Altogether, we conclude from these findings that in the CNS, sodium via ENaC causes EO release and EO via the *α*_2_-isoform increases BP. ENaC activity appears to be an important modulator of the responsiveness of this pathway in the CNS. The highly ouabain-sensitive *α*_3_-isoform is highly expressed in neurons, whereas the ouabain-sensitive *α*_2_-isoform is the isoform in glia (Hieber et al. 1991; Herrera et al. 1994). Considering the presence of the *α*_2_-isoform in glia, mechanisms downstream to ENaC and EO appear to depend on glia signaling. Sympatho-excitatory and pressor responses to central sodium, ouabain and EO also depend on Angiotensin II Type 1 receptor (AT_1_R) stimulation (Huang and Leenen 1996). Since components of the renin-angiotensin system are also present in glia (Milsted et al. 1990; Füchtbauer et al. 2011), and their up- or down regulation increases/decreases BP (Morimoto et al. 2001, 2002) one may speculate that EO increases Ang II release by glia, acting on AT_1_R in glia and/or neurons.

We previously showed that increased ENaC expression in the brain of Nedd4-2^−/−^ mice plays a major role in the hypertension by high-salt diet in these mice by causing an increase in CSF[Na^+^] and increasing responsiveness to CSF sodium (Van Huysse et al. 2012). Blockade of ENaC in the CNS by icv infusion of benzamil prevents most of the high-salt diet-induced hypertension in Nedd4-2^−/−^ mice (Van Huysse et al. 2012). The present study shows that EO binding to the ouabain-sensitive *α*_2_-isoform appears to be downstream to enhanced ENaC activity, as high-salt diet causes only a modest increase in BP in the 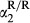/Nedd4-2^−/−^ mice. Altogether, these findings would suggest that the ENaC–EO–*α*_2_-isoform pathway in the CNS plays a major role in the high-salt-induced hypertension in mice with increased ENaC activity. Both the Nedd4-2^−/−^ and the 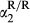 are systemic and ENaC and the *α*_2_-isoform are also present in the kidneys and arteries. The presence of the 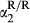 in the kidneys of Nedd4-2^−/−^ mice could influence sodium re-absorption, if plasma EO contributes to regulation of renal function in this model. In arteries, an EO–*α*_2_-isoform signaling cascade may mediate vasoconstriction in response to CNS activation (Blaustein et al. 2012). This response would be blocked in 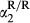 mice (Dostanic et al. 2005). The Nedd4-2^−/−^ mice may also exhibit enhanced ENaC expression in endothelial and smooth muscle cells in arteries (Drummond et al. 2008; Pérez et al. 2009), which may contribute to high-salt-induced myogenic constriction (Jernigan et al. 2008) and enhance vasoconstriction to increased sympathetic tone. The possible role of both these mechanisms in arteries for the high-salt-induced hypertension in the Nedd4-2^−/−^ mice requires further study.

The above described pattern of responses appears specific for salt/CSF [Na^+^], since neither Nedd4-2^−/−^ nor the 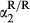 – isoform affected the mild/moderate hypertension induced by sc infusion of Ang II at 200 and 1000 ng/kg/min. In rats, activation of an MR – ENaC – EO pathway in the hypothalamus plays a major role in both salt and Ang II-induced hypertension (Gabor and Leenen 2012). There is also substantial evidence for a critical role of CNS pathways for Ang II-induced hypertension in mice (Zimmerman et al. 2004; Young et al. 2012). The present results would suggest that in mice the MR – ENaC – EO pathway is important for salt/CSF [Na^+^]-induced hypertension but not for Ang II-induced hypertension as neither an increase in plasma membrane ENaC nor blockade of EO actions by the 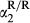 affected the Ang II-induced hypertension.

### Limitation of studies

As a possible limitation of the Nedd4-2^−/−^ mouse as a model for Liddle syndrome, one should consider that Nedd4-2 also regulates other proteins in the brain, such as neuronal voltage-gated sodium channels (Yang and Kumar 2010). Upregulation of these channels in the brain may potentially contribute to the salt-induced hypertension in the ^−/−^ mice, but if so, most unlikely would be blocked by the presence of the 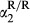 –isoform. Secondly, pressor responses to icv infusion of Na^+^-rich aCSF could have been caused by a volume-induced increase in intracranial pressure or an increase in osmolality. This is very unlikely since infusion of aCSF at the same rate or of mannitol at equivalent osmolality does not increase BP (Van Huysse et al. 2012).

Thirdly, we did not confirm that in mice with combined 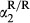/Nedd4-2^−/−^, enhanced ENaC expression on the plasma membrane is still present as is the case for Nedd4-2^−/−^ per se (Van Huysse et al. 2012). Finally, sample sizes of 4–6 mice/group may appear small. However, assessment of BP by telemetry markedly increases the reliability of individual independent values, and these sample sizes for this type of studies are rather common (e.g., Zimmerman et al. 2004; Young et al. 2012).

In conclusion, this study shows that dietary salt-induced hypertension in this mouse model of Liddle syndrome appears to depend on enhanced ENaC–EO–*α*_2_-isoform of Na^+^, K^+^-ATPase signaling. Considering also the increase in CSF[Na^+^] in Nedd4-2^−/−^ mice on high salt (Van Huysse et al. 2012), the absence of pressor responses to CSF[Na^+^] in 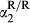/Nedd4-2^−/−^ mice (present study), and the prevention of most of the salt-induced hypertension in Nedd4-2^−/−^ mice by central infusion of benzamil (Van Huysse et al. 2012), inhibition of the *α*_2_-isoform by EO in glia in the CNS appears to play a critical role. Any major role for renal and arterial ENaC appears to depend on these CNS mechanisms.
